# Genetic deletion of amphiregulin restores the normal skin phenotype in a mouse model of the human skin disease tylosis

**DOI:** 10.1242/bio.026260

**Published:** 2017-06-27

**Authors:** Vishnu Hosur, Benjamin E. Low, Leonard D. Shultz, Michael V. Wiles

**Affiliations:** The Jackson Laboratory, Bar Harbor, ME 04609, USA

**Keywords:** CRISPR/Cas9, EGFR, RHBDF2, Amphiregulin, Tylosis

## Abstract

In humans, gain-of-function (GOF) mutations in RHBDF2 cause the skin disease tylosis. We generated a mouse model of human tylosis and show that GOF mutations in RHBDF2 cause tylosis by enhancing the amount of amphiregulin (AREG) secretion. Furthermore, we show that genetic disruption of AREG ameliorates skin pathology in mice carrying the human tylosis disease mutation. Collectively, our data suggest that RHBDF2 plays a critical role in regulating EGFR signaling and its downstream events, including development of tylosis, by facilitating enhanced secretion of AREG. Thus, targeting AREG could have therapeutic benefit in the treatment of tylosis.

## Abstract (precis)

**Summary:** Gain-of-function mutations in RHBDF2 cause tylosis, a skin disease characterized by hyperproliferation of keratinocytes. We generated mice carrying the human tylosis disease mutation p.P189L and show that enhanced amphiregulin secretion underlies tylosis.

## INTRODUCTION

The role of RHBDF2 in enhancing amphiregulin (AREG) secretion, and consequently activating the epidermal growth factor receptor (EGFR) pathway, has significance for the skin disease tylosis. In humans, autosomal dominant mutations in *RHBDF2* cause tylosis (Blaydon et al., 2012; Saarinen et al., 2012), a form of focal nonepidermolytic palmoplantar keratoderma (PPK) that manifests on the skin of the palms and soles (Stevens et al., 1996). Tylosis patients also present oral leukokeratosis and follicular hyperkeratosis (Field et al., 1997; Hennies et al., 1995). Currently, there is no cure for tylosis. In a study to identify genetic variants underlying tylosis, Blaydon et al. used targeted capture array and next-generation sequencing and identified two heterozygous missense mutations in the *RHBDF2* gene that underlie tylosis, p.Ile186Thr and p.Pro189Leu (Blaydon et al., 2012). Further, substantial evidence implicates the involvement of AREG-induced constitutive activation of the EGFR pathway in tylosis (Blaydon et al., 2012; Brooke et al., 2014; Hosur et al., 2014). In addition, skin biopsies of tylosis patients suggest increased EGFR activity (Blaydon et al., 2012). Together, these results, along with the recent findings that mutations in *Rhbdf2* in mice result in enhanced EGFR pathway activation (Hosur et al., 2014; Siggs et al., 2014), suggest a possible mechanism for the association between *RHBDF2* mutations and tylosis*.* However, the detailed physiological role of RHBDF2 in facilitating secretion of AREG needs to be established.

Here, we generated mice carrying a human GOF mutation p.P189L (p.P159L in mice) in *RHBDF2* to examine whether GOF mutations in human RHBDF2 lead to enhanced AREG secretion and tylosis. We provide evidence that *Rhbdf2* GOF mutations enhance AREG secretion to cause hyperplasia and hyperkeratosis. In addition, we show that genetic ablation of AREG attenuates skin disease in the *Rhbdf2^P159L/P159L^* mouse model of human tylosis. Together, our data strongly suggest that inhibition of AREG could have potential therapeutic value in the treatment of tylosis.

## RESULTS AND DISCUSSION

### The human tylosis disease mutation enhances AREG secretion

**Fig. 1.**
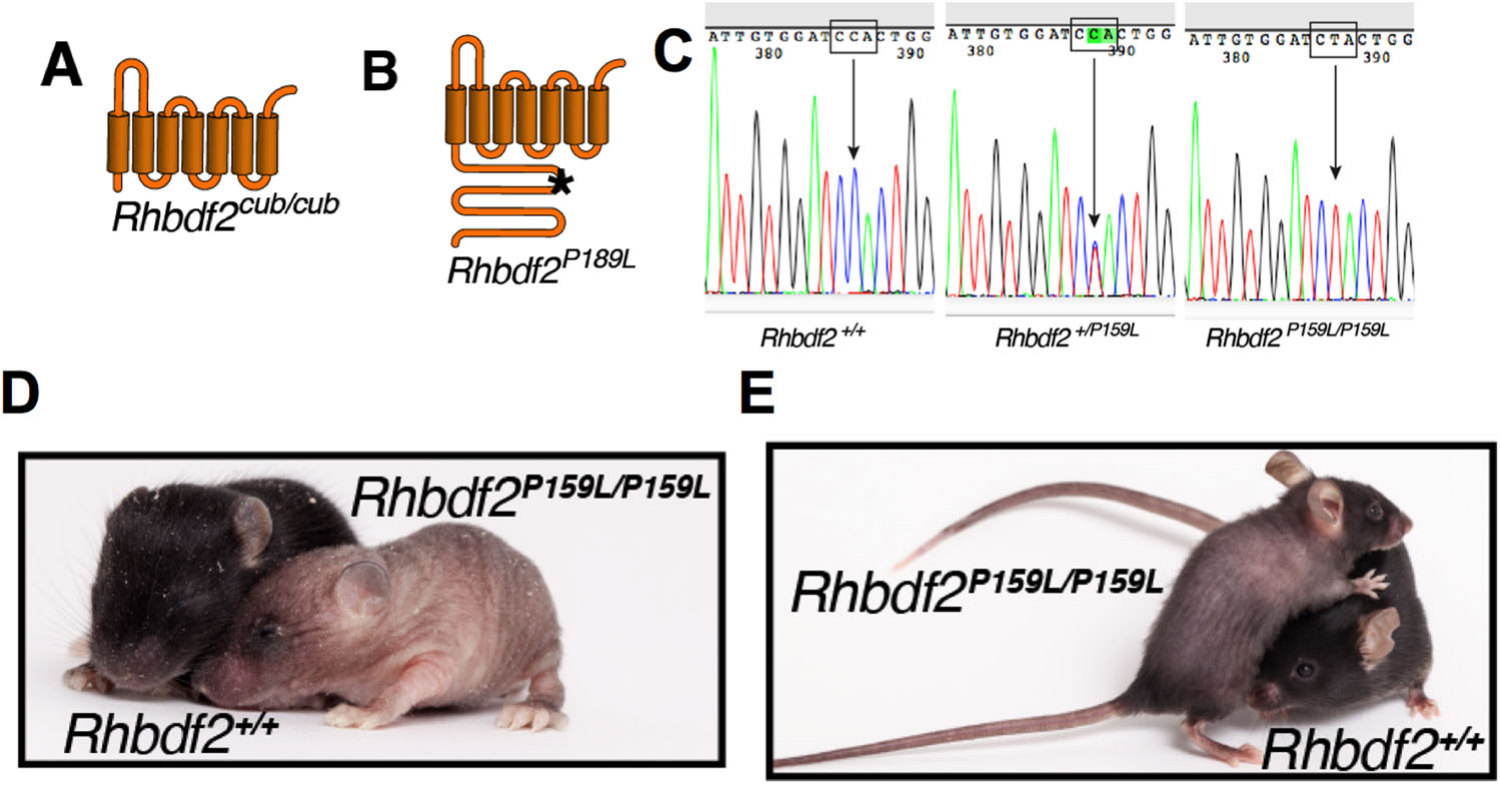
**The human tylosis mutation enhances secretion of AREG.** (A) Schematic of the mouse *Rhbdf2^cub/cub^* mutation, which results in the loss of the cytosolic N-terminus domain of RHBDF2 (ΔN-RHBDF2). (B) Schematic of the human *RHBDF2* (p.P189L) mutation in the cytosolic N-terminus domain. (C) Sequencing chromatograms of *Rhbdf2^+/+^*, *Rhbdf2^+/ P159L^* and *Rhbdf2 ^P159L/P159L^* mice. Arrows indicate a heterozygous single nucleotide polymorphism (SNP) (CCA/CTA) or homozygous SNP (CTA). (D) Images of *Rhbdf2^+/+^* and *Rhbdf2^P159L/P159L^* mice at postnatal day 11. Significant hair loss is visible in *Rhbdf2 ^P159L/ P159L^* mice as early as day 11. (E) Representative image of an eight-week-old female *Rhbdf2^P159L/P159L^* mouse presenting a thin hair coat.

**Fig. 2.**
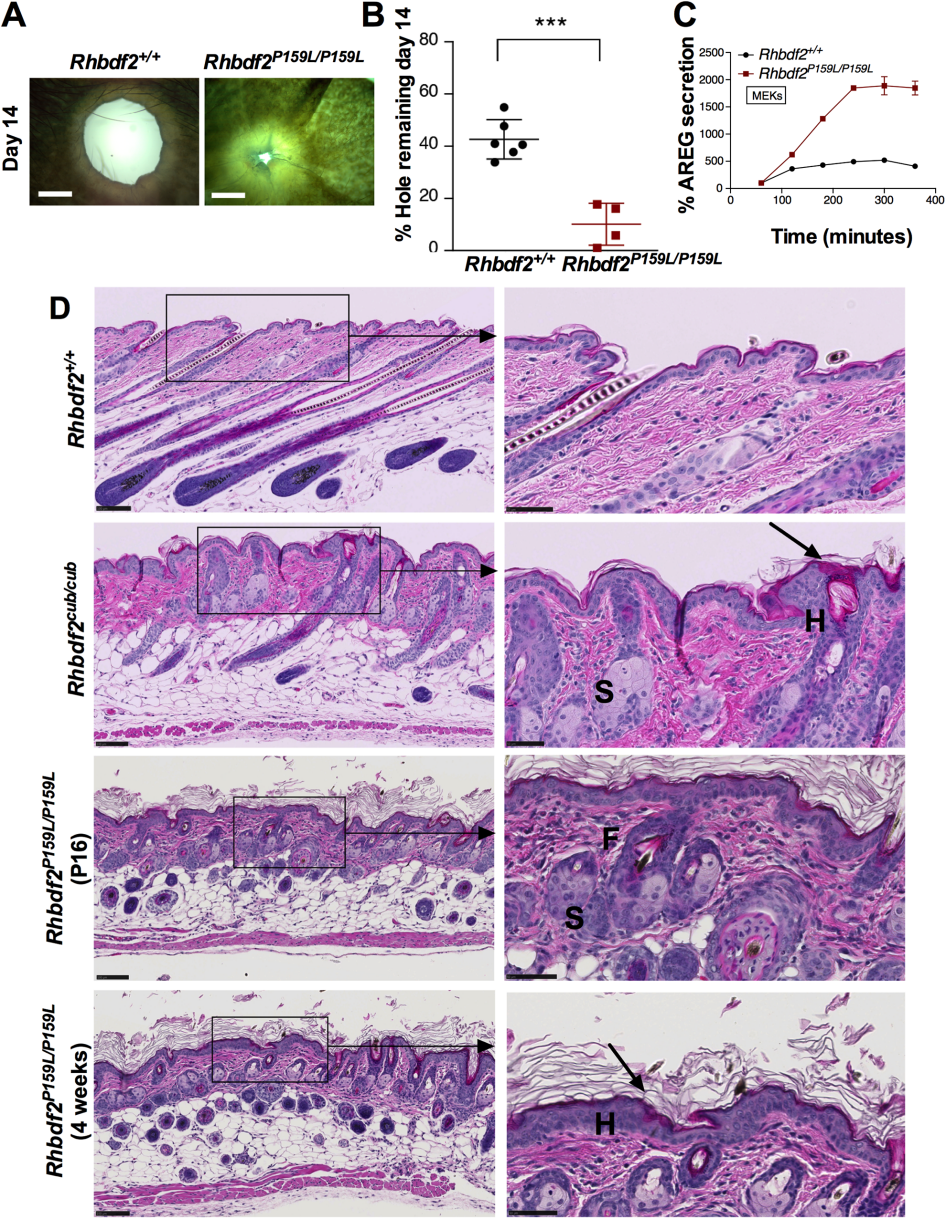
**Rapid wound healing and proliferative skin phenotype in *Rhbdf2^P159L/P159L^* mice.** (A) Representative images of regenerating ear tissue in eight-week-old female *Rhbdf2^+/+^ and Rhbdf2^P159L/ P159L^* mice at 14 days postwounding. Magnification, 4×; scale bars: 1 mm. (B) Quantification of ear hole closures shown in A. *Rhbdf2^+/+^* (*n*=6) and *Rhbdf2^P159L/P159L^* (*n*=4). (C) ELISA quantitation of AREG levels in the supernatants of cultured *Rhbdf2^+/+^ and Rhbdf2^P159L/P159L^* MEKs in response to stimulation with 100 nM PMA for the indicated times. (D) H&E-stained skin sections of female *Rhbdf2^+/+^, Rhbdf2^cub/cub^* and *Rhbdf2^P159L/P159L^* mice*.* Both the *Rhbdf2^cub/cub^ and Rhbdf2^P159L/P159L^* mice present with significant hyperplasia (H), hyperkeratosis (arrow), enlarged sebaceous glands (S), and follicular dystrophy (F) compared with the *Rhbdf2^+/+^* mice. Scale bars: 100 μm (low magnification); 50 μm (high magnification).

Autosomal dominant mutations in the human *RHBDF2* gene cause tylosis (Blaydon et al., 2012; Saarinen et al., 2012). Substantial evidence implicates the involvement of AREG-induced constitutive activation of the EGFR pathway (Blaydon et al., 2012; Brooke et al., 2014; Hosur et al., 2014) in human tylosis. We recently showed that similar to GOF mutations in the mouse *Rhbdf2* gene (*Rhbdf2^cub/cub^*), GOF mutations in human *RHBDF2* (tylosis mutation p.I186T) promote increased secretion of AREG *in vitro* (Hosur et al., 2014). To determine *in vivo* whether the *Rhbdf2^cub^* mutation (Fig. 1A, loss of the cytosolic N-terminal domain of RHBDF2) phenocopies human tylosis, and whether the tylosis p.P189L mutation (Fig. 1B, missense mutation in the cytosolic N-terminal domain of RHBDF2) in *RHBDF2* increases AREG secretion, we generated mice carrying the human tylosis mutation p.P189L (p.P159L in mice) using CRISPR/Cas9-mediated targeting and homology-directed repair in C57BL/6J zygotes (Fig. 1C). We characterized the resulting *Rhbdf2^P159L/P159L^* mice and observed that similarly to *Rhbdf2^cub/cub^* mice (Hosur et al., 2014), mice carrying the human tylosis mutation present with complete hair loss (Fig. 1D). However, unlike *Rhbdf2^cub/cub^* mice (Hosur et al., 2014; Siggs et al., 2014), which present complete hair loss throughout their lives, *Rhbdf2^P159L/P159L^* mice developed a thin layer of curly hair coat that was noticeable in adult mice (Fig. 1E), indicating that the *Rhbdf2^cub^* phenotype is more severe than that of *Rhbdf2^P159L^*. Nevertheless, *Rhbdf2^P159L/P159L^* mice elicited significantly accelerated wound healing, as determined by ear punch closure (Fig. 2A,B). Additionally, accelerated wound healing was associated with an increase in stimulated secretion of AREG in *Rhbdf2^P159L/P159L^* MEKs compared with *Rhbdf2^+/+^* MEKs (Fig. 2C).

**Fig. 3.**
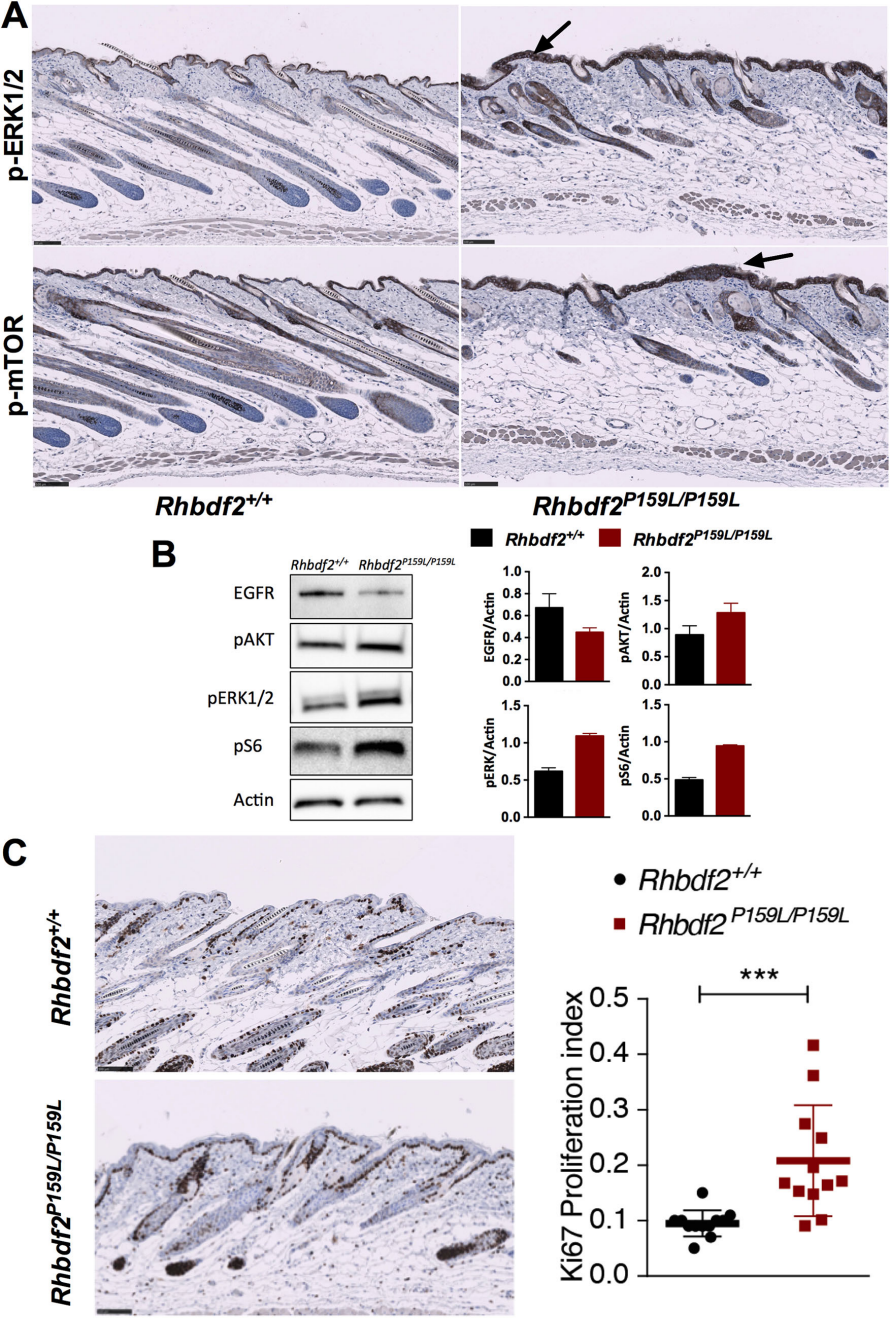
**Enhanced EGFR signaling in *Rhbdf2^P159L/P159L^* mice.** (A) Immunohistochemical staining of *Rhbdf2^+/+^ and Rhbdf2^P159L/P159L^* skin sections with phospho-ERK1/2 and phospho-mTOR. Arrows indicate increased levels of phospho-ERK1/2 and phospho-mTOR staining. Scale bars: 100 μm. (B) Immunoblot analysis of cell lysates obtained from *Rhbdf2^+/+^* and *Rhbdf2^P159L/P159L^* MEFs. Actin served as a loading control. Band intensities were quantified using ImageJ (https://imagej.nih.gov/ij/). (C) Immunohistochemical staining of *Rhbdf2^+/+^* and *Rhbdf2^P159L/P159L^* skin sections with proliferation marker Ki-67. Quantification of proliferating cells was performed as described previously (Almeida et al., 2012). Scale bars: 100 μm.

Next, to examine whether mice carrying the human tylosis mutation phenocopy the *Rhbdf2^cub^* mutation (Hosur et al., 2014), we performed histological analysis of skin sections from *Rhbdf2^+/+^* and *Rhbdf2^P159L/P159L^* mice. *Rhbdf2^P159L/P159L^* mice presented with follicular dystrophy (F), enlarged sebaceous glands (S), hyperplasia (H), and hyperkeratosis (arrow) of skin (Fig. 2D). To assess enhanced EGFR signaling in the skin of *Rhbdf2^P159L/P159L^* mice, we performed immunohistochemical analyses of skin sections from *Rhbdf2^+/+^* and *Rhbdf2^P159L/P159L^* mice. We observed a considerable increase in epidermal EGFR signaling pathway downstream effectors phospho-ERK1/2 and phospho-mTOR (Fig. 3A). Similarly, immunoblot analyses of mouse embryonic fibroblasts (MEFs) isolated from *Rhbdf2^+/+^* and *Rhbdf2^P159L/P159L^* mice also revealed enhanced EGFR signaling as demonstrated by reduced EGFR protein levels (due to downregulation of sustained EGFR receptor activation), and by enhanced protein levels of phospho-AKT, phospho-ERK1/2 and phospho-S6 in *Rhbdf2^P159L/P159L^* MEFs compared with those of *Rhbdf2^+/+^* mice (Fig. 3B). Also, there was a two- to threefold increase in the levels of the proliferation marker Ki-67 in the skin of *Rhbdf2^P159L/P159L^* mice compared with that of *Rhbdf2^+/+^* mice (Fig. 3C). Collectively, these data suggest that *Rhbdf2^cub/cub^* and *Rhbdf2^P159L/P159L^* are GOF mutations in the *Rhbdf2* gene, and that these mutations enhance secretion of AREG to cause tylosis.

### AREG deficiency restores the normal skin phenotype in *Rhbdf2^P159L/P159L^* mice

**Fig. 4.**
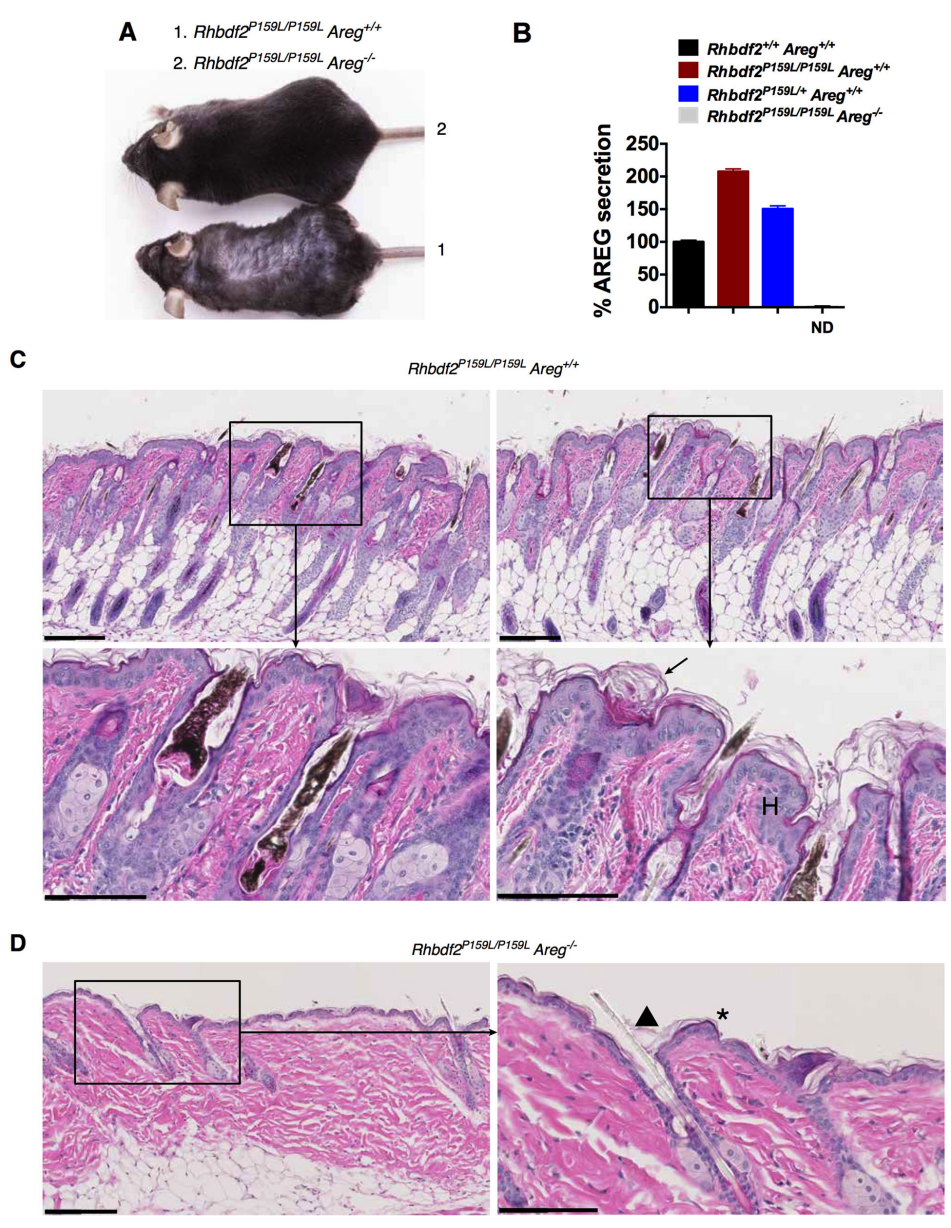
**Genetic deletion of *Areg* restores the normal skin phenotype in *Rhbdf2^P159L/P159L^* mice.** (A) Representative images of age-matched *Rhbdf2^P159L/P159L^* and *Rhbdf2^P159L/P159L^ Areg^−/−^* male mice. *Rhbdf2^P159L/ P159L^* mice show a loss of hair phenotype (1); *Rhbdf2^P159L/P159L^ Areg^−/−^* mice show a full hair coat (2). (B) Percentage serum AREG levels in age-matched female *Rhbdf2^+/+^*, *Rhbdf2^P159L/+^*, *Rhbdf2^P159L/P159L^* and *Rhbdf2^P159L/ P159L^ Areg^−/−^* mice. AREG was not detected (ND) in the serum of *Rhbdf2^P159L/P159L^ Areg^−/−^* mice. Data represent mean±s.d. (*n*=4 mice per group). (C,D) H&E-stained skin sections of male *Rhbdf2^P159L/P159L^* and *Rhbdf2^P159L/ P159L^ Areg^−/−^* mice. Follicular dystrophy, hyperplasia (H) and hyperkeratosis (arrow) observed in *Rhbdf2^P159L/P159L^* mice are restored in *Rhbdf2^P159L/P159L^ Areg^−/−^* mice. We found no evidence of follicular dystrophy (arrowhead), hyperplasia or hyperkeratosis of skin (asterisk) in *Rhbdf2^P159L/P159L^ Areg^−/−^* mice. Scale bars: 200 μm (low magnification); 100 μm (high magnification).

Mice carrying the human tylosis GOF mutation *Rhbdf2^P159L^* elicit enhanced AREG secretion (Fig. 2). To test whether AREG drives the skin disease phenotype in *Rhbdf2^P159L/P159L^* mice, we genetically deleted AREG in *Rhbdf2^P159L/P159L^* mice by crossing *Rhbdf2^P159L/P159L^* with AREG-null mice. *Rhbdf2^P159L/P159L^* mice lacking AREG presented a full wavy coat rather than a partial hair-loss phenotype of *Rhbdf2^P159L/P159L^* mice (Fig. 4A). We confirmed that these mice are null for AREG by measuring serum AREG levels in *Rhbdf2^+/+^*, *Rhbdf2^P159L/+^*, *Rhbdf2^P159L/P159L^* and *Rhbdf2^P159L/P159L^ Areg^−/−^* mice. As expected, we found no detectable levels of AREG in *Rhbdf2^P159L/P159L^ Areg^−/−^* mice (Fig. 4B). Additionally, whereas homozygous mutant *Rhbdf2^P159L/P159L^* mice had various degrees of follicular dystrophy or hair loss, the homozygous mutant mice with no AREG had no follicular dystrophy or hair loss (Fig. 4C). Also, there was no evidence of hyperplasia or hyperkeratosis of skin in *Rhbdf2^P159L/P159L^ Areg^−/−^* mice (Fig. 4D). Collectively, these data strongly suggest that AREG mediates the skin disease phenotype of mice carrying the human tylosis mutation *Rhbdf2^P159L^*, and that AREG is thus a potential therapeutic target for treating the skin disease tylosis.

## MATERIALS AND METHODS

### Animals

Mice were bred and maintained under modified barrier conditions at The Jackson Laboratory (JAX). The Animal Care and Use Committee at JAX approved the experimental procedures. For the tylosis mutation, we targeted the *Rhbdf2* locus in C57BL/6J zygotes by pronuclear microinjection of Cas9 mRNA (60 ng/μl), single guide RNA (sgRNA) (30 ng/μl), and single-stranded oligonucleotide DNA (ssDNA) (1 ng/μl). A total of 10 pups were born, and five of the 10 pups exhibited partial to complete hair loss. Tail DNA was amplified using PCR and then sequenced, and subsequently founder mice carrying the desired modified alleles were backcrossed to C57BL/6J mice to validate germline transmission. The resulting offspring heterozygous for the *Rhbdf2^P159L^* allele were intercrossed to generate homozygous C57BL/6J-*Rhbdf2^P159L/P159L^* mice. Similar to the *Rhbdf2^cub/+^* heterozygous mice (Hosur et al., 2014; Johnson et al., 2003; Siggs et al., 2014), the *Rhbdf2^P159L/+^* heterozygous mice present with a normal hair coat, and moreover there is no evidence of epidermal hyperplasia or hyperkeratosis. We recently showed that B6.Cg-*Areg^Mcub^ Rhbdf2^cub^*/J mice carry a T-to-G point mutation that disrupts the coding frame and introduces a premature stop codon in the *Areg* gene (Hosur et al., 2014). B6.Cg-*Areg^Mcub^ Rhbdf2^cub^*/J do not produce *a* functional AREG protein; hence, *Areg^Mcub/Mcub^* mice are referred to as *Areg^−/−^* in this manuscript.

### Ear hole closure and histology

Ear punch closure, histology and histological analyses of hematoxylin and eosin (H&E)-stained sections were performed as previously described (Hosur et al., 2014). Slides were scanned using a Nanozoomer digital slide scanner (2.0-HT, Hamamatsu Photonics, Hamamatsu, Japan) and analyzed with the help of Dr Rosalinda Doty, a board-certified pathologist at JAX.

### Isolation of fibroblasts and keratinocytes

MEFs were isolated from 13.5 days postcoitum mouse embryos using Liberase DL (Sigma-Aldrich). Embryos were dissected in 15 ml sterile serum-free DMEM and minced with a razor blade in a 100 mm petri dish. The DMEM medium was replaced with fresh serum-free DMEM containing Liberase DL (0.5 mg/ml), and the cell suspension was allowed to incubate at 37°C for 75 min. Following incubation, cells were centrifuged, washed three times with sterile PBS, re-suspended in DMEM containing 10% fetal bovine serum and antibiotic/antimycotic (Thermo Fisher Scientific), and plated in collagen-coated T-75 flasks. Mouse embryonic keratinocytes were isolated from the skin of embryonic day 18 mouse embryos using Neutral Protease (Dispase) (Worthington Biochemical Corporation, Lakewood, USA). Briefly, skin was peeled off, washed twice with sterile PBS, and incubated in Keratinocyte Growth Medium-2 (Lonza, Allendale, USA) containing antibiotic/antimycotic (Thermo Fisher Scientific). Following overnight incubation at 4°C, epidermis was carefully separated from dermis and placed in a sterile petri dish containing 2 ml trypsin (TrypLE Select Enzyme, Thermo Fisher Scientific) for 30 min at room temperature. Subsequently, trypsin was neutralized with Defined Trypsin Inhibitor (Thermo Fisher Scientific), the cell suspension was centrifuged, and cells were seeded in collagen-coated six-well plates.

### AREG ELISA

MEKs seeded in collagen-coated six-well dishes were stimulated with 100 nM PMA (R&D Systems, Minneapolis, USA) for the indicated times. AREG protein levels in the conditioned medium were measured using a Mouse Amphiregulin DuoSet ELISA Developmental Kit (#DY989, R&D Systems). A spectrophotometer (SpectraMax 190, Molecular Devices, Sunnyvale, USA) was used to determine the optical density.

### SDS/PAGE and immunoblotting

SDS/PAGE and immunoblotting assays were performed as described previously (Hosur et al., 2014). Briefly, MEFs grown in six-well dishes were lysed with RIPA buffer (Cell Signaling Technology) and protein concentrations were determined using a Qubit Fluorometer (Life Technologies). After loading 50 μg of protein onto 4-20% (wt/vol) precast gels (Lonza), proteins were transferred to a PVDF membrane, before blocking with 5% milk for 1 h at room temperature (RT). Membranes were then exposed to EGFR (#2232; 1:1000), phospho-ERK1/2 (#4370; 1:1000), phospho-AKT (#4060; 1:1000), phospho-S6 (#4858,; 1:1000 dilution) or actin (#4970; 1:1000) antibodies (Cell Signaling Technology) overnight at 4°C. Subsequently, membranes were washed in TBST for 2 h, exposed to secondary antibodies (Santa Cruz Biotechnology; 1:10,000) for 1 h at RT, and washed for 2 h in TBST. Membranes were then exposed to Luminol reagent (Santa Cruz Biotechnology) for 3 min and visualized using a gel documentation system (G:BOX F3, Syngene, Frederick, USA).

### Immunohistochemistry

Immunohistochemical staining of skin sections with specific antibodies [phospho-p44/42 MAPK (ERK1/2) (#4370, Cell Signaling Technology; 1:400), phospho-mTOR (#2976, Cell Signaling Technology; 1:100) and Ki-67 (Thermo Fisher Scientific; prediluted)] was performed on a Ventana Discovery XT automated IHC research slide staining system (Roche, Tucson, USA). For antigen unmasking, deparaffinized slides were pretreated with Cell Conditioning Solution (Ki-67) or citrate buffer (10 mM Sodium Citrate, 0.05% Tween 20, pH 6.0) (phospho-ERK1/2 and phospho-mTOR). Ki-67 proliferation index was determined as described previously (Almeida et al., 2012).

### Statistical analysis

GraphPad Prism v6 was used to analyze data. Student's *t*-test was used to assess the statistical difference between two groups. *P*<0.05 was considered statistically significant.
